# Ecotoxicity of Combined Polylactic Acid Microplastics and Thallium Pollution on the Functional Traits of *Folsomia candida*

**DOI:** 10.3390/toxics14040307

**Published:** 2026-04-02

**Authors:** Yuying Chen, Guoliang Xu, Zhijian Wu, Cao Hao, Chen Yang, Xiaohua Chen

**Affiliations:** 1 School of Geography and Remote Sensing, Guangzhou University, Guangzhou 510006, China; chenyy@e.gzhu.edu.cn (Y.C.); 2112301033@e.gzhu.edu.cn (Z.W.); haoc107@163.com (C.H.); 2112501022@e.gzhu.edu.cn (C.Y.); chenxhua@gzhu.edu.cn (X.C.); 2 Rural Non-Point Source Pollution Comprehensive Management Technology Center of Guangdong Province, Guangzhou 510006, China

**Keywords:** polylactic acid microplastics (PLA-MPs), thallium (Tl), *Folsomia candida*, functional traits, gut bacterial community

## Abstract

Microplastics can bind with toxic metals via surface complexation and chelation, forming combined pollutants. However, research regarding the toxicological impacts of these combined pollutants on soil fauna remains limited. This study employed *Folsomia candida* in a 28-day incubation experiment to investigate the ecotoxicological effects of combined pollution by polylactic acid microplastics (PLA-MPs) and thallium (Tl) on the functional traits of *Folsomia candida*, including biology, morphology, and gut microbiota. The results showed that the combined effects of PLA-MPs and Tl on these functional traits were characterized by amplified toxicity and trait-specific responses. Morphological traits exhibited lower sensitivity to the pollution treatments compared to other indicators. Exposure to high-concentration PLA-MPs (10%) significantly affected mortality and fecundity, and reduced gut bacterial diversity. Conversely, low-concentration Tl (1 mg/kg) significantly inhibited body length and antenna length while increasing gut bacterial diversity. Structured equation modeling further revealed that the pollution treatments exerted significant negative effects on the functional traits of *Folsomia candida*, both directly and indirectly by altering soil properties and soil microbiota. These findings provide valuable insights into the ecotoxicological effects of combined PLA-MPs and Tl pollution on soil fauna, contributing to ecological health risk assessments of microplastics and toxic metals in terrestrial ecosystems.

## 1. Introduction

Thallium (Tl) is a highly toxic metal [1], and its long-term accumulation in the environment poses severe threats to ecosystems and human health [2,3]. Anthropogenic activities, such as the exploitation of Tl-rich mineral deposits and the substantial discharge of industrial waste, are major drivers of Tl pollution. These activities release Tl into the surface environment, where it gradually accumulates in the soil [4]. The toxicity of Tl to living organisms far exceeds that of common toxic metals like mercury, cadmium, copper, and lead [5]. Studies have demonstrated that Tl can inhibit microbial activity, reduce microbial abundance [6], and impair the growth and development of animal bones, leading to delayed embryonic development or morphological abnormalities [7]. In addition, Tl can be absorbed by humans through the skin and mucous membranes, bioaccumulating in the bones, kidneys, and central nervous system, causing severe, potentially fatal health complications [8]. Therefore, Tl is classified as one of the 13 priority toxic metal pollutants by the U.S. Environmental Protection Agency [9] and is included in China’s Priority Control Chemicals List [10].

Recently, the interaction between toxic metals and microplastics, an emerging class of pollutants, has garnered widespread scientific attention, particularly regarding their combined ecological impacts on soil ecosystems [11,12,13]. Microplastics not only negatively affect plants, soil fauna, and microorganisms, but they also pose potential threats to human health via trophic transfer along the food chain [14,15,16]. Their large specific surface area facilitates the adsorption of toxic pollutants in the environment, including toxic metals, polycyclic aromatic hydrocarbons (PAHs), and polychlorinated biphenyls (PCBs) [17]. Once adsorbed, microplastics can induce a “Trojan horse” effect, acting as vectors that alter the migration behavior and bioavailability of these pollutants. This can result in synergistic or antagonistic effects on soil fauna [18,19]. For example, combined exposure to polystyrene microplastics and cadmium has been shown to cause severe oxidative stress, metabolic inhibition, and DNA damage in earthworms [20]. However, current research on the toxicological effects of microplastics on soil fauna predominantly focused on traditional, non-degradable polymers. Studies investigating the toxicity of biodegradable microplastics such as polylactic acid (PLA), polyhydroxybutyrate, and polypropylene carbonate on collembola remain scarce [21], leaving their underlying ecotoxicological mechanisms largely unclear.

Collembola are widely used as model organisms in ecotoxicological research to evaluate soil pollution levels and assess ecological health risks [22]. As one of the three major groups of soil fauna [23], collembola play important roles in soil development, nutrient cycling, and the maintenance of biotic communities [24]. Furthermore, they exhibit pronounced sensitivity to environmental pollutants. Functional traits are measurable characteristics of organisms, including morphology, physiology, phenology, and behavior [25], and directly reflect a species’ adaptation to its environment and heavily influence various ecosystem functions [26,27]. For instance, collembolan body size serves as an indicator of food resource utilization efficiency, predation capacity, and dispersal ability [28]. Similarly, the furca can reflect habitat preferences, as species with a well-developed furca typically favor grasslands over forest environments [29]. Additionally, the gut bacterial community, which co-evolves with the host [30], is shaped by multiple environmental factors. It directly affects host survival and adaptability by participating in physiological processes, such as nutrient decomposition, metabolite synthesis, and immune defense [31]. Currently, research employing functional traits to evaluate the ecotoxicological effects of soil pollutants on collembola is quite scarce.

Therefore, this study utilized the model organism *Folsomia candida* in a 28-day microcosm incubation experiment. By employing microscope observation, qPCR, and high-throughput sequencing technologies, we systematically investigated the changes in the functional traits of *F. candida* under single and combined exposures to varying concentrations of PLA-MPs and Tl from the perspectives of biological traits, morphological traits, and bacterial communities. Additionally, we analyzed the complex relationships between pollution treatments, soil environment, and *F. candida* functional traits. These findings aim to enrich the toxicological database regarding the combined effects of PLA-MPs and Tl on soil microorganisms and fauna, ultimately contributing to more accurate ecological health risk assessments for microplastics and toxic metals in terrestrial environments.

## 2. Materials and Methods

### 2.1. Materials

Thallium nitrate (TlNO_3_, Shanghai Aladdin Biochemical Technology Co., Ltd., Shanghai, China) was stored at 4 °C and diluted with ultrapure water to achieve the required concentrations. PLA-MPs were sourced from Hengfa Plasticization (Dongguan, China). Previous studies, such as those by Crouau Y et al. [22], indicate that *F. candida* preferentially ingests microplastic particles ≤50 µm. Consequently, PLA-MPs with a particle size of approximately 48 µm (capable of passing through a 300-mesh sieve) were selected for the exposure experiments. Before use, the PLA-MPs were sterilized with a 70% ethanol rinse (Guangzhou Chemical Reagent Factory, Guangzhou, China), washed twice with deionized water, and dried at 40 °C. Microbial DNA was extracted using the FastDNA™ Spin Kit for Soil (MP Biomedicals, Irvine, CA, USA). The experimental substrate was an artificial soil prepared according to OECD guidelines [32], consisting of sphagnum peat (Pindstrup Matrix Official Store, Linyi, China), kaolin clay (Henan Platinum Run Casting Materials Co., Ltd., Zhengzhou, China), and quartz sand (Kainuo Trading Co., Ltd., Hebi, China) in a 1:2:7 mass ratio. The soil pH was adjusted to 6.0 ± 0.5 using acetic acid (CH_3_COOH, Guangdong Xinchengyuan Technology Co., Ltd., Huizhou, China), and the moisture content was adjusted to 50% of its maximum water-holding capacity using ultrapure water. This study utilized *F. candida* cultured in a controlled laboratory climate chamber. They were reared in culture vessels containing a 0.5 cm basal layer of a plaster of Paris and activated charcoal mixture at a mass ratio of 9:1 [33]. Active dry yeast was provided as food. The environmental conditions were strictly maintained at 20 ± 1 °C with a 12:12 h light/dark cycle and a relative humidity of 65–70%. Ultrapure water and fresh yeast were replenished every 3 days, during which any moldy food residues and dead *F. candida* were promptly removed to maintain optimal culture conditions. To eliminate potential biases arising from age and size variations, age-synchronized *F. candida* were prepared before the experiment. Approximately 200 active, similarly sized adult individuals were transferred to fresh culture vessels, supplied with dry yeast, and maintained under standard conditions. After observing that *F. candida* had laid eggs, all adult individuals were removed after 48 h. The eggs were left to incubate for 13–15 days. Upon the hatching of the majority of the eggs, any remaining unhatched eggs were discarded, yielding an age-synchronized *F. candida* for the formal toxicological experiment.

### 2.2. Experimental Design

A 28-day microcosm incubation experiment was conducted, exposing *F. candida* directly to artificial soil with different concentrations of polylactic acid microplastics (PLA-MPs) and Thallium (Tl). Based on prior ecotoxicological studies regarding biodegradable microplastics and their environmental accumulation levels [21], the PLA-MP concentrations were established at 0, 0.1%, 1%, and 10% (*w*:*w*). Concurrently, Tl concentrations were set at 0, 1, and 10 mg/kg. These specific doses were selected to reflect typical background Tl pollution levels in industrial and mining soils [4,5,6], and were further validated by preliminary acute toxicity tests on *F. candida*. The experimental design comprised a full factorial setup with 12 distinct treatments (Table 1), each performed with 5 replicates, yielding a total of 60 microcosms. PLA-MPs particles and TlNO_3_ solution were incrementally homogenized into the artificial soil. In the combined pollution groups, PLA-MPs were incorporated first, followed by TlNO_3_ solution to ensure homogeneous distribution. The prepared contaminated soil was then pre-incubated in the artificial climate chamber for a 7-day aging period to facilitate adsorption equilibrium between PLA-MPs, Tl, and the soil matrix. Soil moisture was maintained by periodically supplementing with ultrapure water during this period.

Following the aging process, 30 g aliquots of the contaminated soil were distributed into 100 mL glass beakers, and 20 age-synchronized *F. candida* juveniles were introduced into each beaker along with a small provision of dry yeast. The beakers were sealed with perforated plastic film to prevent the escape of *F. candida*, while permitting gas exchange, and subsequently incubated in the climate chamber for 28 days. Ultrapure water and yeast were supplemented every 3 days. At the end of the experiment, the soil was flooded with ultrapure water to float *F. candida*. Surviving adults and newly hatched larvae were collected for morphological trait measurements and gut microbiome profiling. Additionally, representative soil samples from each replicate were collected: one subsample was used for physicochemical analysis, while another was immediately frozen at −20 °C for soil bacterial community sequencing.

### 2.3. Measurement Methods

Evaluated soil physicochemical properties included pH, organic matter (OM), ammonium nitrogen (NH_4_^+^-N), nitrate nitrogen (NO_3_^−^-N), available phosphorus (AP), and available potassium (AK). Soil pH was measured using a standard glass electrode pH meter. Soil OM was determined by the potassium dichromate oxidation method with external heating. NH_4_^+^-N and NO_3_^−^-N were measured using the indophenol blue colorimetric method and the dual-wavelength colorimetric method, respectively. AP was determined by the molybdenum antimony anti-colorimetric method, and AK was quantified using flame photometry. Anhydrous ethanol was used to fix the morphology of *F. candida*. Fixed samples were imaged using a Leica S8AP0 stereomicroscope (Leica Microsystems GmbH, Wetzlar, Germany). The number of surviving adults and newly hatched larvae under different treatments was counted. ImageJ 1.54g software was used to measure the body length of all adults and over half of the larvae, as well as the antenna and furca lengths of adults.

Total genomic DNA was extracted from the soil and *F. candida* gut samples using the FastDNA™ Spin Kit for Soil. High-throughput amplicon sequencing of the microbiota was outsourced to Shanghai Majorbio Bio-Pharm Technology Co., Ltd. (Shanghai, China). The V3-V4 hypervariable region of the bacterial 16S rRNA gene was targeted for PCR amplification using the universal primer pair 338F (5′-ACTCCTACGGGAGGCAGCAG-3′) and 806R (5′-GGACTACHVGGGTWTCTAAT-3′). Following purification and quantification of the PCR amplicons, sequencing libraries were constructed, and paired-end sequencing was conducted on an Illumina MiSeq platform (Illumina, CA, USA). Raw paired-end reads were merged based on overlapping regions and subjected to stringent quality control and filtering. The high-quality sequences were then clustered into Operational Taxonomic Units (OTUs) at a 97% sequence similarity threshold, with chimeric sequences identified and removed. To normalize variations in sequencing depth for downstream alpha and beta diversity analyses, all samples were rarefied to a uniform depth, achieving an average sequence coverage of 99.09%. Taxonomic annotation of the OTUs was performed using the RDP Classifier against the Silva 16S rRNA database (v138) at a confidence threshold of 70%, allowing for the determination of community composition across various taxonomic ranks [34,35].

### 2.4. Statistical Analysis

All statistical analyses were conducted using SPSS statistical software, version 26.0. A one-way analysis of variance (ANOVA) was employed to assess statistical differences among the various pollution treatments. Where significant main effects were detected, Tukey’s Honestly Significant Difference (HSD) post-hoc test was applied for multiple comparisons. Statistical significance was defined as *p* < 0.05, and disparate lowercase letters were used to denote significant inter-treatment differences. Graphical representations of the biological and morphological trait data were generated using Origin 2021. Furthermore, visual analyses of bacterial community diversity, taxonomic composition, and assembly processes were conducted on the Majorbio Cloud Platform (https://cloud.majorbio.com, format: accessed on 4 November 2025). To quantify the magnitude of responses, weighted effect sizes and corresponding 95% confidence intervals (CIs) for biological traits, morphological parameters, and bacterial diversity indices were calculated using a fixed-effects model via the rmamv function in the “metafor” package in R, version 4.5.1 [36]. Partial Least Squares Path Modeling (PLS-PM) was constructed using the “plspm” package, version 0.6.0 [37], and the model was further optimized based on significance (*p* < 0.05) and goodness of fit (GOF > 0.6).

## 3. Results

### 3.1. Effects of PLA-MPs and Tl on Soil Properties

The effects of PLA-MPs and Tl on soil physicochemical properties are shown in Table 2. Significant differences in soil pH were observed across the different pollution treatments (*p* < 0.05). Compared to the control (CK), pH in the P_3_, P_3_T_1_, and P_3_T_2_ treatments decreased by 7.66%, 18.31%, and 18.37%, respectively. OM content also differed significantly (*p* < 0.05). It increased by 35.37%, 41.07%, and 53.91% in the P_3_, P_3_T_1_, and P_3_T_2_ treatments compared to the CK, while reaching its lowest level in the T_2_ treatment. In contrast, NH_4_^+^-N content showed no significant differences among treatments, with slight increases observed exclusively in the T_1_ and P_3_T_2_ treatments. NO_3_^−^-N content varied significantly (*p* < 0.05). It was elevated in the P_3_, P_3_T_1_, and P_3_T_2_ treatments compared to the CK, rising concurrently with PLA-MPs concentrations, and further intensifying under combined pollution treatments. Furthermore, both AP and AK contents showed significant differences among treatments (*p* < 0.05). Compared to the CK, both AP and AK levels exhibited a dose-dependent decrease with increasing PLA-MP concentrations, a trend that was exacerbated under combined pollution treatments. Overall, PLA-MPs exerted a dominant influence on soil properties. High-concentration PLA-MPs significantly reduced pH, AP, and AK contents while increasing OM and NO_3_^−^-N contents. The interactive effect of Tl primarily manifested as an exacerbation of soil acidification and a partial mitigation of specific nutrient alterations.

### 3.2. Significance Analysis of Pollution Treatments on the Functional Traits of F. candida

Two-way ANOVA was performed to show the main effects of PLA-MPs and Tl on the functional traits of *F. candida* (Table 3). Significant interactive effects between PLA-MPs and Tl were detected for mortality, fecundity, and both soil and gut bacterial Chao1 indices (*p* < 0.001), as well as antenna length (*p* < 0.01). These results indicated that the combined effects of the two pollutants were not simply additive, but exhibited trait-specific synergistic or antagonistic interactions.

Subsequently, t-tests were used to compare the differences between each treatment and the control (CK) (Table 4). Exposure to high-concentration PLA-MPs significantly affected the biological and morphological traits of *F. candida*, as well as the diversity of both soil and gut bacterial communities. Biological traits demonstrated high sensitivity to pollution treatments, while antenna length was the only morphological trait to show a clear response across all treatments. Furthermore, most treatments exerted significant effects on the gut bacterial community diversity.

**Figure 1:**
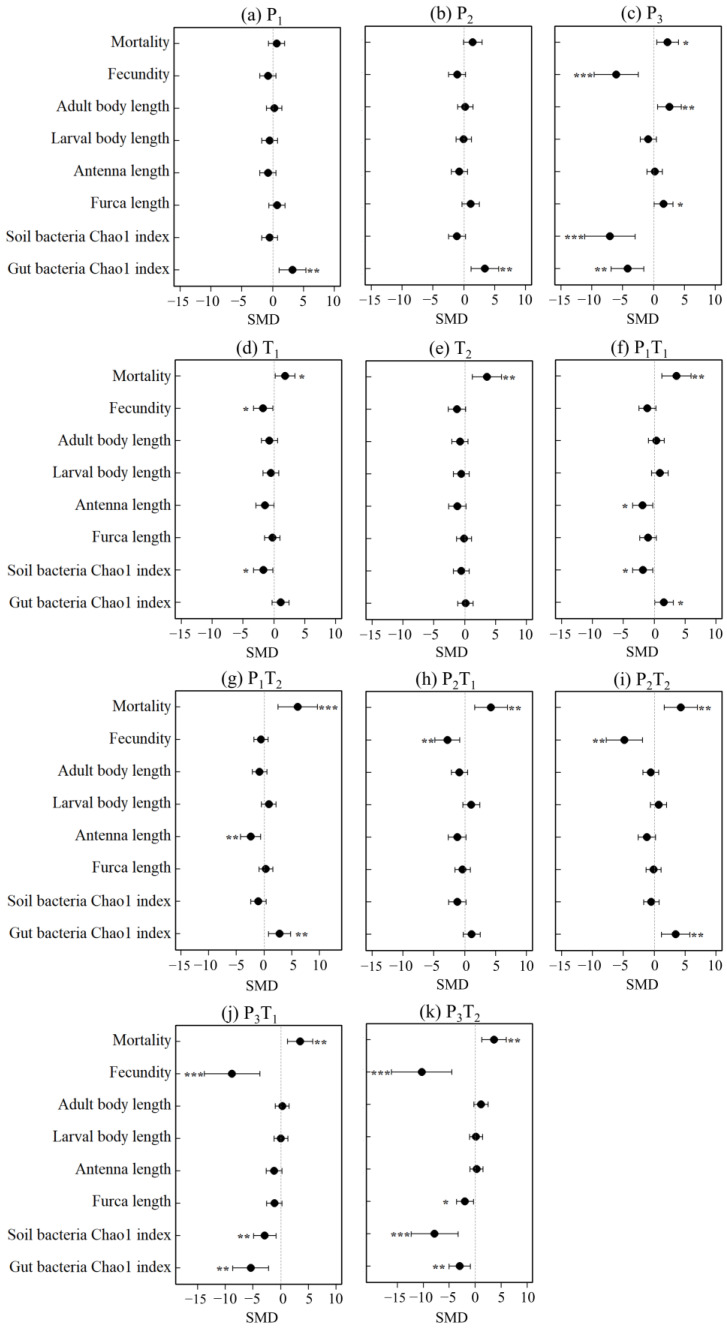
Weighted effect sizes (standardized mean difference, SMD) with 95% confidence intervals (CIs) for each functional trait of *F. candida* under different treatments. Effect sizes were calculated using a fixed-effects model. Positive and negative SMD values indicated promotion and inhibition of the trait, respectively, relative to the control (CK). (**a**–**k**) denote different pollution treatments. * 0.01 < *p* < 0.05; ** 0.001 < *p* < 0.01; *** *p* < 0.001.

The response magnitude for each functional trait of *F. candida* varied significantly across pollution treatments (Figure 1). The P_3_ treatment showed the most pronounced overall impact, significantly altering all evaluated traits with the exception of larval body length and antenna length. Within this group, fecundity and the soil bacterial Chao1 index exhibited the greatest deviations, underscoring the severe solitary effect of high-concentration PLA-MPs. Under the P_3_T_2_ treatment, the deviations in fecundity and the soil bacterial Chao1 index surpassed those observed in the P_3_ and T_2_ treatments, reflecting a robust synergistic regulatory effect of high-concentration combined pollution on *F. candida* functional traits. Interestingly, the P_3_T_1_ treatment yielded less pronounced effects on adult body length, furca length, and the soil bacterial Chao1 index compared to the P_3_ treatment. This suggests an antagonistic interaction, wherein low-concentration Tl partially alleviated the ecotoxicity of high-concentration PLA-MPs on specific morphological parameters and microbiomes. Overall, while high-concentration PLA-MPs profoundly disrupted biological traits and bacterial diversity, morphological traits (excluding antenna length) remained comparatively resilient to the pollution treatments.

### 3.3. Effects of PLA-MPs and Tl on Biological Traits of F. candida

**Figure 2:**
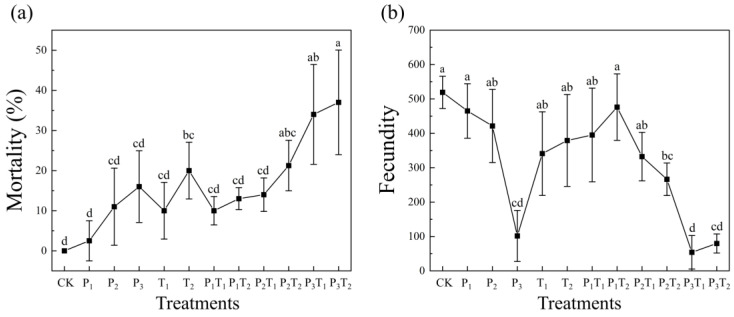
The effects of different pollution treatments on (**a**) mortality and (**b**) fecundity of *F. candida*. Different lowercase letters indicated significant differences between pollution treatments (Tukey’s HSD test, *p* < 0.05).

Following the 28-day exposure period, *F. candida* mortality differed significantly among pollution treatments (*p* < 0.05). Elevated mortality was recorded across all experimental groups compared to the CK, demonstrating a discernible dose–response pattern (Figure 2a). Mortality showed an upward trajectory, peaking at over 35% in the P_3_T_1_ and P_3_T_2_ treatments, significantly higher than in all other cohorts. Notably, mortality in the P_1_T_2_ treatment was lower than that in the T_2_ treatment, indicating that low-concentration PLA-MPs alleviated the toxicity of high-concentration Tl. Similarly, fecundity varied significantly among treatments (*p* < 0.05). As illustrated in Figure 2b, fecundity was significantly decreased in the P_3_ treatment and reached its lowest levels in the P_3_T_1_ and P_3_T_2_ treatments, representing a 90% reduction compared to the CK. These data indicate a negative correlation between fecundity and PLA-MP concentration; conversely, Tl exerted no discernible impact on fecundity. However, the co-exposure of medium to high concentrations of PLA-MPs alongside Tl severely exacerbated this reproductive inhibition, confirming that high-concentration PLA-MPs act as the primary driver of reduced fecundity.

### 3.4. Effects of PLA-MPs and Tl on Morphological Traits of F. candida

**Figure 3:**
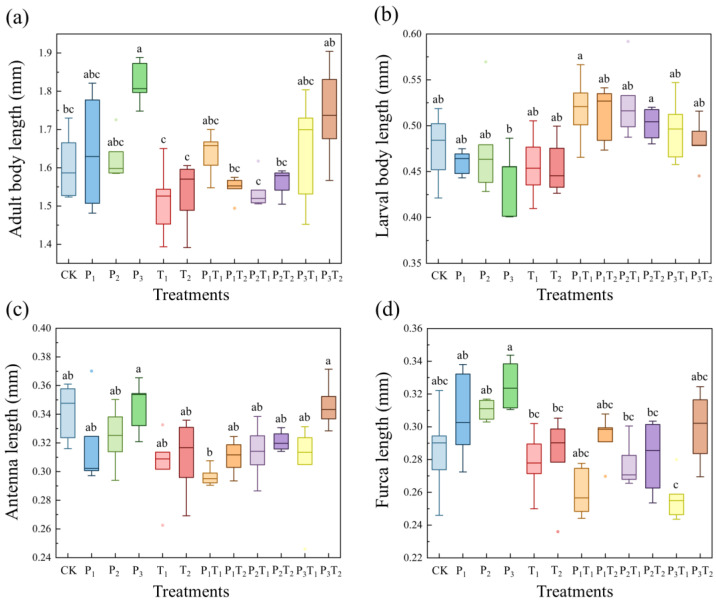
The effects of various pollution treatments on (**a**) adult body length, (**b**) larval body length, (**c**) antenna length, and (**d**) furca length of *F. candida*. Different lowercase letters indicated significant differences between pollution treatments (Tukey’s HSD test, *p* < 0.05).

Morphological traits, including adult body length, larval body length, antenna length, and furca length, all exhibited statistically significant differences across the pollution treatments (*p* < 0.05). As shown in Figure 3a, compared to the CK, exposure to PLA-MPs alone increased adult body length, achieving a maximum length increase of 13.45% in the P_3_ treatment. Conversely, solitary Tl inhibited adult body length. Interestingly, combined pollution treatments alleviated the inhibitory effect of Tl on adult body length. Figure 3b showed that both individual PLA-MPs and Tl exposures significantly reduced larval body length, with the most severe stunting occurring in the P_3_ treatment; however, combined exposures again mitigated the Tl-induced inhibition. PLA-MPs showed no discernible impact on antenna length, while Tl exposure caused significant shortening (Figure 3c). As observed with body length, combined pollution treatments alleviated this Tl-induced antenna inhibition. Figure 3d showed that PLA-MPs alone obviously increased furca length. In summary, while high-concentration PLA-MPs significantly promoted adult body and furca length, Tl acted primarily as a growth inhibitor, stunting adult body length, larval body length, and antenna length.

### 3.5. Effects of PLA-MPs and Tl on the Gut Bacterial Community of F. candida and the Soil Bacterial Community

**Figure 4:**
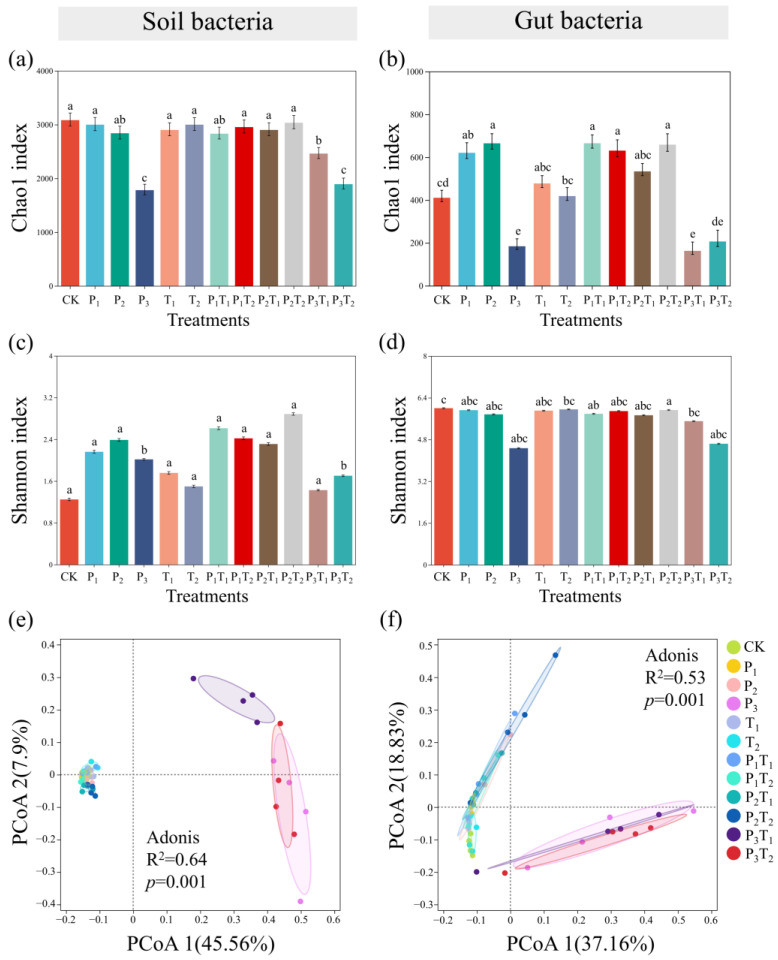
The effects of different pollution treatments on the alpha diversity of (**a**,**c**) soil and (**b**,**d**) *F. candida* gut bacterial communities. Different lowercase letters indicated significant differences between pollution treatments (Tukey’s HSD test, *p* < 0.05). Principal Coordinate Analysis (PCoA) based on Bray–Curtis distance was used to analyze changes in the bacterial community structure of (**e**) soil and (**f**) *F. candida* gut. Adonis (PERMANOVA) analysis was performed to test significant differences in community structure among treatments.

Microenvironmental shifts induced by the pollution treatments significantly altered the diversity and taxonomic composition of bacterial communities [38]. Analysis of alpha diversity revealed that, compared to the CK, Chao1 and Shannon indices of the soil bacterial community significantly decreased only in the P_3_, P_3_T_1_, and P_3_T_2_ treatments, hitting their absolute minimums in the P_3_ treatment (Figure 4a,c). Correspondingly, the Chao1 index of *F. candida* gut bacterial community was substantially depressed in these same high PLA-MPs treatments (Figure 4b,d). Principal Coordinate Analysis (PCoA) demonstrated spatial overlap among the gut bacterial community in the P_3_, P_3_T_1_, and P_3_T_2_ treatments (Figure 4f), underscoring that high-concentration PLA-MPs dictate community structure, albeit with a more severe disruptive effect observed in the soil matrix than within the gut.

**Figure 5:**
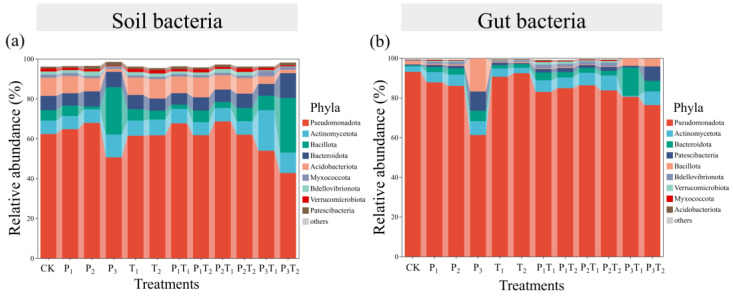
Bacterial community composition of (**a**) soil and (**b**) *F. candida* gut under different pollution treatments. Phyla with relative abundance >1% are shown.

Taxonomic profiling at the phylum level further elucidated these compositional shifts. Pseudomonadota dominated both the soil and gut bacterial communities, followed by Actinobacteria. Compared to the CK, the relative abundance of soil Pseudomonadota in P_3_, P_3_T_1_, and P_3_T_2_ treatments decreased by 11.69%, 8.38%, and 19.57%, respectively, while the abundance of Bacillota increased by 18.53%, 2.09%, and 22.18% in these respective groups (Figure 5a). Within the gut, Pseudomonadota populations experienced even steeper declines of 31.84%, 12.56%, and 16.72% in the P_3_, P_3_T_1_, and P_3_T_2_ treatments, respectively. Conversely, Actinobacteriota increased by 4.79%, 4.23%, and 4.08% in the P_2_T_2_, P_3_, and P_3_T_2_ treatments, while Patescibacteria increased by 9.31% and 6.87% in the P_3_ and P_3_T_2_ treatments (Figure 5b). Ultimately, high-concentration PLA-MPs had acted as the primary driver of microbial dysbiosis in both environments. Tl enhanced the effect of PLA-MPs on the soil bacterial community but weakened its impact on the gut bacterial community.

**Figure 6:**
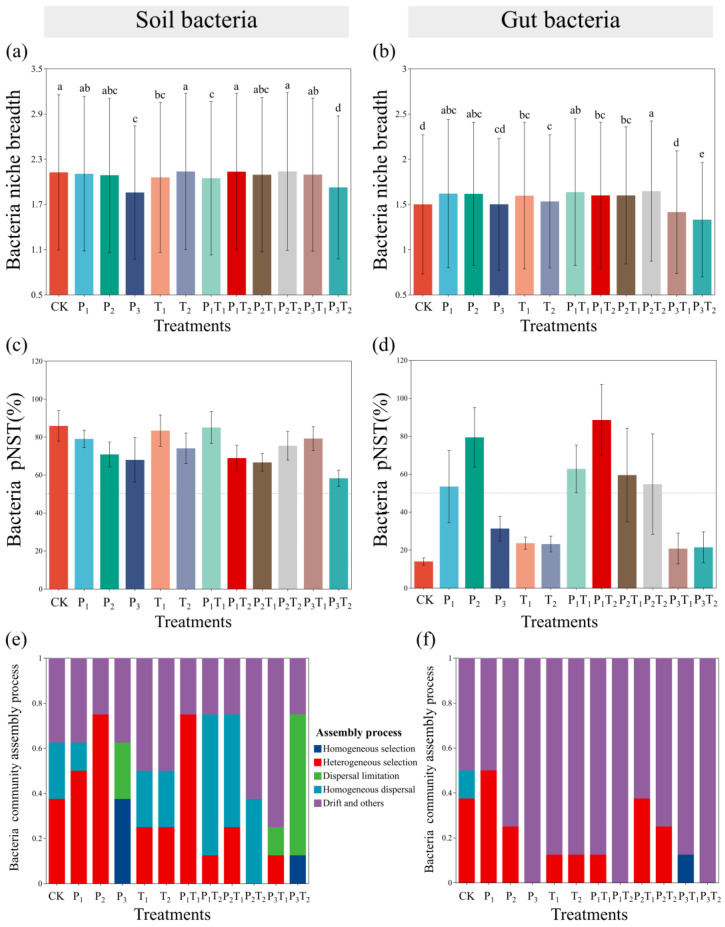
Ecological niche shifts in (**a**) soil and (**b**) *F. candida* gut bacteria, different lowercase letters indicated significant differences between pollution treatments (Tukey’s HSD test, *p* < 0.05). Ecological stochasticity in the assembly of (**c**) soil and (**d**) gut bacterial communities was estimated by the phylogenetic normalized stochasticity ratio (pNST) based on Bray–Curtis distance. The community assembly processes of (**e**) soil and (**f**) gut bacteria were indicated by the βNTI and RCbray indices.

The ecological niche of bacterial communities under different pollution treatments showed a consistent pattern, with only the P_3_ and P_3_T_2_ treatments causing a significant contraction of niche breadth in both soil and gut bacterial communities (Figure 6). Such a narrowed niche breadth typically signals constrained resource utilization and diminished environmental adaptability [39]. High-concentration PLA-MPs severely disrupted normal bacterial metabolism, thereby undermining community stability. Applying the null model frameworks developed by Stegen et al. [40] and Ning et al. [41], we evaluated the underlying mechanisms of community assembly. Soil bacterial assembly under all treatments was dominated by stochastic processes (Figure 6c). In contrast, gut bacterial community assembly shifted from deterministic to stochastic process dominance in the P_1_, P_2_, P_1_T_1_, P_1_T_2_, P_2_T_1_, and P_2_T_2_ treatments, indicating that low (0.1%) to medium (1%) concentrations of PLA-MPs strongly amplified the role of stochasticity in gut colonization (Figure 6d). In addition, the P_1_, P_2_, and P_1_T_1_ treatments elevated the influence of heterogeneous selection within the soil, while the P_3_, P_3_T_1_, and P_3_T_2_ treatments suppressed heterogeneous selection in favor of homogeneous selection and dispersal limitation (Figure 6e). Gut bacterial community assembly was governed primarily by ecological drift and heterogeneous selection. Notably, while the P_1_ treatment bolstered heterogeneous selection, drift became the exclusive, overriding assembly mechanism in the P_3_, P_1_T_2_, and P_3_T_2_ treatments (Figure 6f).

### 3.6. Relationships Between Pollution Treatments, Soil Environment, and Functional Traits of F. candida

**Figure 7:**
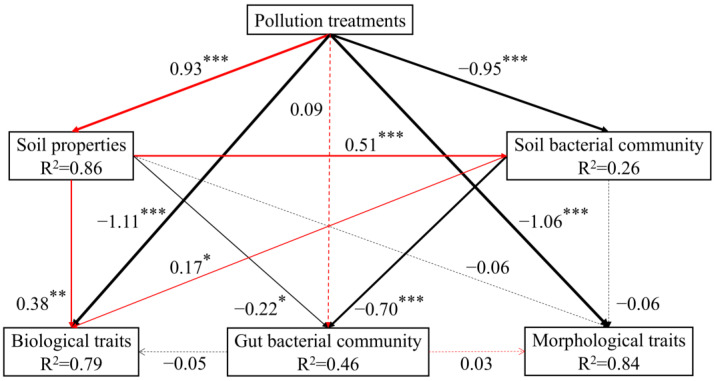
PLS-PM shows the direct and indirect effects of pollution treatments, soil environment, and functional traits of *F. candida*. The red solid lines and black solid lines indicate positive and negative relationships, respectively. The dashed lines represent non-significant path coefficients. The numbers next to the arrows are standardized path coefficients, and the width of the arrow represents the strength of the path coefficients. The R^2^ values indicate the proportion of explained variance. * *p* < 0.05, ** *p* < 0.01 and *** *p* < 0.001.

Partial Least Squares Path Modeling (PLS-PM) was utilized to disentangle the complex direct and indirect relationships connecting the pollution treatments, the soil environment (properties and microbiota), and the functional traits of *F. candida* (biology, morphology, and gut microbiota) (Figure 7). The constructed model accounted for 79%, 84%, and 46% of the variance observed found in the biological traits, morphological traits, and gut bacterial community, respectively. Additionally, it explained 86% and 26% in the soil properties and soil bacterial community, respectively. Path analysis revealed that the pollution treatments exerted profound direct negative effects on both biological (λ = −1.11, *p* < 0.001) and morphological traits (λ = −1.06, *p* < 0.001). Shifts within the gut bacterial community were significantly and directly governed by alterations in soil properties (λ = −0.22, *p* < 0.05) and the soil bacterial community (λ = −0.70, *p* < 0.001). Consequently, because the pollution treatments fundamentally restructured soil properties (λ = 0.93, *p* < 0.001) and decimated the soil bacterial community (λ = −0.95, *p* < 0.001), they wielded a powerful indirect influence over the gut bacterial community. In synthesis, the combined PLA-MPs and Tl pollution degraded the overall functional traits of *F. candida* via a dual mechanism: immediate direct toxicity and indirect environmental mediation.

## 4. Discussion

### 4.1. Effects of Pollution Treatments on the Functional Traits of F. candida

This study elucidated the toxicological effects of combined pollution on the functional traits of *F. candida*, highlighting amplified combined effects and trait-specific responses. Two-way ANOVA revealed significant interactive effects between PLA-MPs and Tl on multiple functional traits of *F. candida*, confirming that the combined effects of the two pollutants cannot be equated to the sum of their individual effects. Exposure to high-concentration PLA-MPs emerged as the primary driver altering these functional traits, significantly impacting mortality, fecundity, and bacterial diversity (Figure 1). These findings align with previous research demonstrating that elevated microplastic concentrations significantly impair collembolan reproduction and gut microbiota homeostasis. [42]. Interestingly, high-concentration PLA-MPs had no significant effect on larval body length and antenna length, possibly due to the protective role of chitinous exoskeleton, which dampens the sensitivity of morphological traits to external stressors [43,44]. Furthermore, Tl did not induce the classic hormetic (low-dose stimulation, high-dose inhibition) response typically associated with toxic metals [45]. Low-concentration Tl exhibited only marginal effects on mortality, fecundity, and soil bacterial diversity, while high-concentration Tl significantly affected only mortality (Figure 1). This deviation is attributed to the extreme acute toxicity of Tl, even low environmental concentrations surpass the physiological tolerance threshold of *F. candida* [6,7]. Under severe Tl stress, *F. candida* appears to adopt a stress-responsive survival strategy characterized by the drastic reallocation of energy and physiological resources. Specifically, the organism prioritizes limited endogenous energy for fundamental survival processes, such as cellular respiration and basal metabolism, while reducing or even suspending investment in non-essential functions like reproduction and somatic growth [46]. This may also stem from the relatively short experimental duration, as 28 days may not be sufficient to fully reflect the cumulative toxic effects of high-concentration Tl on fecundity and development.

The toxicological impacts of combined pollution were predominantly synergistic, reinforcing the hypothesis that microplastics act as vectors that exacerbate the toxicity of toxic metals [47]. However, low-concentration Tl alleviated the toxicity of high-concentration PLA-MPs on specific morphological traits and bacterial diversity, suggesting a potential antagonistic interaction or “toxicity competition”. The ecotoxicity of biodegradable microplastics is primarily associated with cellular damage caused by their degradation byproducts [48]. It is plausible that low-concentration toxic metals interact with these degradation products, thereby reducing the overall bioavailability of the microplastics [49]. The responses of *F. candida* functional traits were highly specific. Fecundity and bacterial diversity are directly related to population viability [50] and stress adaptability to pollution stress, as well as the maintenance of ecological functionality [51], thus becoming priority response indicators. In contrast, traits such as body length, antenna length, and furca length remained largely unaffected across most treatments. Beyond the physical barrier provided by the exoskeleton, this resilience likely reflects an evolutionary energy allocation strategy that prioritizes the maintenance of essential morphological structures to ensure basic survival. This aligns with broader evidence indicating that collembolan morphological traits possess robust tolerance to environmental disturbances [29]. In summary, this study establishes fecundity and microbiome diversity as the premier sentinels of pollution stress in *F. candida*, clarifying the synergistic nature of combined pollution and furnishing a quantitative foundation for future soil ecological risk assessments.

### 4.2. Effects of Pollution Treatments on Biology, Morphology, and Gut Bacterial Community of F. candida

Both the biological (Figure 2) and morphological traits (Figure 3) of *F. candida* showed significant dose-dependent responses and interactive effects of pollutants. The ingestion of microplastics by soil fauna can induce severe physical trauma and tissue inflammation, which subsequently impairs growth, suppresses fecundity, and elevates mortality risk [52]. Overall, high-concentration PLA-MPs exerted the most profound detrimental impacts on the mortality, fecundity, and morphology of *F. candida*, corroborating previous toxicological models [53]. Similarly, under metal ion stress, collembolan survival and developmental metrics demonstrate distinct dose–response relationships [46]. In this study, even low-concentration Tl severely inhibited the growth, development, and fecundity of *F. candida*. This contrasts sharply with literature showing that low concentrations of essential metals like Cu can promote collembolan growth [45], while non-essential toxic metals like Cd suppress fecundity primarily at high concentrations [54]. This discrepancy highlights the intense, idiosyncratic toxicity of Tl compared to other metals. Combined pollution exhibited synergistic toxicity on *F. candida* biology and morphology. Microplastics bind toxic metals via surface complexation and chelation, forming highly bioavailable composite pollutants that dramatically amplify ecological toxicity risks [17].

Microorganisms serve as vital sentinels of environmental change, driving nutrient cycling and energy flux within soil ecosystems [55]. Concurrently, the host-associated microbiome, which co-evolves with soil fauna, is indispensable for regulating host development, immunity, and environmental adaptation [56]. This study found that high-concentration PLA-MPs significantly collapsed the diversity of both the soil and gut bacterial communities (Figure 4). This dysbiosis is likely driven by microplastic-induced alterations to soil physicochemical properties and available resource pools [16]. PLA-MPs enriched Bacillota while inhibiting Pseudomonadota, whereas Tl favored Bacteroidota at the expense of Bacillota (Figure 5). We hypothesize that PLA-MPs compromise the collembolan gut barrier, triggering inflammatory cascades while simultaneously acting as adsorption sinks for Tl^+^. This localized accumulation of composite pollutants likely eradicates sensitive bacterial taxa while selecting for highly stress-tolerant species [57]. Furthermore, Tl could weaken the disturbance of PLA-MPs on the gut bacterial community, possibly because the binding of Tl^+^ to functional groups on the microplastic surface neutralizes reactive sites, thereby mitigating the direct physical and chemical trauma inflicted by the particles on bacterial cells [58]. Ecological niche breadth reflects the environmental adaptation or resource utilization capacity of a species or population [59]. High-concentration PLA-MPs significantly narrowed the bacterial ecological niche breadth (Figure 6), indicating weakened environmental adaptability and resource utilization capacity. Microbial community assembly is jointly driven by stochastic and deterministic processes [60]. Exposure to low-concentration and medium-concentration PLA-MPs triggered a transition from deterministic to stochastic dominance, driven primarily by heterogeneous selection and ecological drift (Figure 6). Degradation byproducts released from the microplastics likely precipitated this assembly shift [61]. Drift, which dominates in destabilized or shrinking populations, is a primary catalyst for biodiversity loss [62], while heterogeneous selection drives compositional divergence among microbial communities [63].

### 4.3. Relationships Between Treatments, Soil Environment, and Functional Traits of F. candida

By employing PLS-PM, this study mapped the intricate interplay among the pollution treatments, the soil environment, and *F. candida* functional traits, revealing a dual-pathway regulatory mechanism composed of direct toxicity and environment mediation. The pollution treatments exerted severe, direct negative effects on both biological and morphological traits. However, the regulatory influence of soil properties was highly divergent: while soil parameters negatively influenced morphological traits and gut bacterial diversity, they exerted a significant positive buffering effect on biological traits (Figure 7). It is speculated that soil properties inhibit morphological traits and the gut bacterial community by altering the bioavailability of pollutants [64,65]. Conversely, soil properties positively support fecundity by adsorbing and fixing pollutants and optimizing nutrient supply, thereby prioritizing the core function of population continuation [66,67]. Crucially, the soil environment functioned as the primary mediator dictating gut microbiome health. The pollution treatments indirectly degraded the gut bacterial community by initially devastating soil properties and the free-living soil microbiome (Figure 7). Previous studies have demonstrated that shifting soil conditions directly degrade the nutritional quality of food resources of collembola. Declines in soil microbial diversity disrupt critical carbohydrate and energy metabolism [68], leading to impaired soil nutrient transformation processes [69]. This cascading nutritional deficit starves *F. candida*, inducing gut dysbiosis and subsequently compromising nutrient absorption and innate immune defenses [70,71]. The core mechanism of this study involves the synergistic effects of physical damage caused by PLA-MPs and the chemical toxicity of Tl. These agents directly assault the cellular integrity and metabolic equilibrium of *F. candida* while indirectly degrading its physiological landscape via soil and microbial deterioration. Under this dual pressure, the functional traits of *F. candida* exhibited significant negative responses. These findings indicated that soil properties and microbial community dynamics are indispensable intermediary indicators for the ecological risk assessment of combined pollution, supplying a robust mechanistic framework for future soil remediation strategies.

## 5. Conclusions

This study thoroughly investigated the individual and combined ecotoxicological effects of PLA-MPs and Tl on the functional traits of *F. candida*. The composite effects of these pollutants were characterized by amplified combined effects and trait-specific responses. High-concentration PLA-MPs acted as the primary driver of mortality and fecundity, while Tl predominantly stunted body length and antenna length. PLA-MPs exacerbated the inhibitory effect of Tl on fecundity but alleviated Tl’s stunting effect on antenna length. Furthermore, while high-concentration PLA-MPs significantly reduced the diversity of both soil and gut bacterial communities, the co-presence of Tl partially attenuated this microplastic-driven dysbiosis. Low and medium concentrations of PLA-MPs shifted gut bacterial community assembly from deterministic to stochastic processes, increasing the contribution of drift and reducing the importance of heterogeneous selection. The combined pollution exerted significant direct toxicity on the biological and morphological traits of *F. candida*, while utilizing soil properties and soil bacterial community as critical intermediary indicators for indirect ecological damage. Ultimately, this study deepened the understanding of how combined PLA-MPs and Tl pollution degrade collembolan functional traits through dual direct–indirect pathways. These insights furnish vital scientific indicators for the risk assessment of complex soil contamination scenarios and establish a theoretical foundation for ecosystem protection and targeted soil remediation. Future research should focus on investigating the specific molecular pathways linking microbiome dysbiosis directly to host phenotypic collapse, a necessary step for developing advanced, targeted soil restoration technologies.

## Figures and Tables

**Table 1:** Different pollution treatments in the experimental design ^1^.

Treatments	PLA-MPs Concentration (*w*:*w*)	Tl Concentration (mg/kg)
CK	0	0
P_1_	0.1%	0
P_2_	1%	0
P_3_	10%	0
T_1_	0	1
T_2_	0	10
P_1_T_1_	0.1%	1
P_1_T_2_	0.1%	10
P_2_T_1_	1%	1
P_2_T_2_	1%	10
P_3_T_1_	10%	1
P_3_T_2_	10%	10

^1^ PLA-MPs: Polylactic acid microplastics; Tl: Thallium.

**Table 2:** Soil properties under different pollution treatments ^1^.

Treatments	pH	OM (g/kg)	NH_4_^+^-N (mg/kg)	NO_3_^−^-N (mg/kg)	AP (mg/kg)	AK (mg/kg)
CK	6.37 c	80.49 c	2.98 a	0.39 c	39.18 a	61.03 a
P_1_	6.75 a	76.07 c	2.27 a	0.26 c	33.82 b	56.27 ab
P_2_	6.47 bc	80.02 c	2.27 a	0.54 c	31.54 bcd	52.16 bc
P_3_	5.88 d	108.96 b	1.55 a	1.42 ab	27.50 d	48.79 cd
T_1_	6.52 bc	73.46 c	3.82 a	0.20 c	31.00 bcd	46.35 cd
T_2_	6.50 bc	71.04 c	2.62 a	0.20 c	32.90 bc	51.23 bc
P_1_T_1_	6.47 bc	76.16 c	1.67 a	0.83 bc	31.12 bcd	47.46 cd
P_1_T_2_	6.62 ab	80.66 c	1.55 a	0.31 c	30.80 bcd	47.86 cd
P_2_T_1_	6.40 bc	77.73 c	1.79 a	0.36 c	29.50 cd	50.87 bcd
P_2_T_2_	6.41 bc	80.53 c	2.86 a	0.23 c	30.14 bcd	45.55 cd
P_3_T_1_	5.20 e	113.55 ab	2.27 a	1.65 a	27.46 d	43.60 d
P_3_T_2_	5.20 e	123.88 a	3.34 a	1.44 ab	28.08 d	45.58 cd

^1^ OM: Organic matter; NH_4_^+^-N: Ammonium nitrogen; NO_3_^−^-N: Nitrate nitrogen; AP: Available phosphorus; AK: Available potassium. Different lowercase letters indicated significant differences between pollution treatments (Tukey’s HSD test, *p* < 0.05).

**Table 3:** Two-way ANOVA for the main effects of PLA-MPs and Tl on the functional traits of *F. candida* ^1^.

Functional Traits	PLA-MPs		Tl		PLA-MPs × Tl	
	F	P	F	P	F	P
Mortality	5.91	0.006	15.00	0.001	15.11	0.000
Fecundity	27.89	0.000	3.79	0.053	28.07	0.000
Adult body length	5.14	0.011	1.44	0.276	3.11	0.019
Larval body length	0.91	0.462	0.49	0.624	1.31	0.289
Antenna length	1.52	0.248	3.31	0.072	4.75	0.002
Furca length	2.99	0.062	0.10	0.906	3.29	0.015
Soil bacterial Chao1 index	36.40	0.000	2.57	0.131	31.57	0.000
Gut bacterial Chao1 index	55.69	0.000	1.85	0.213	18.06	0.000

^1^ F: F-statistic of two-way ANOVA; P: significance level of two-way ANOVA.

**Table 4:** *T*-tests comparing each treatment to the control (CK) on the functional traits of *F. candida* ^1^.

Treatments	Mortality		Fecundity		Adult Body Length		Larval Body Length		Antenna Length		Furca Length		Soil Bacterial Chao1 Index		Gut Bacterial Chao1 Index	
	t	p	t	p	t	p	t	p	t	p	t	p	t	p	t	p
P_1_	−1.00	0.374	1.32	0.224	−0.46	0.657	0.90	0.397	1.36	0.212	−1.22	0.258	0.74	0.487	−5.04	0.002
P_2_	−2.56	0.063	1.88	0.113	−0.44	0.674	0.04	0.969	1.27	0.240	−1.95	0.087	1.72	0.136	−5.34	0.002
P_3_	−4.00	0.016	10.63	0.000	−4.53	0.002	1.43	0.196	−0.34	0.746	−2.82	0.022	11.05	0.000	6.61	0.001
T_1_	−3.16	0.034	3.06	0.027	1.57	0.154	0.81	0.444	2.55	0.034	0.47	0.652	2.71	0.035	−1.73	0.134
T_2_	−6.33	0.003	2.21	0.058	1.34	0.218	0.89	0.409	2.06	0.073	0.21	0.843	0.91	0.399	−0.20	0.845
P_1_T_1_	−6.33	0.000	1.93	0.113	−0.61	0.558	−1.56	0.163	4.63	0.006	1.76	0.117	2.91	0.027	−2.59	0.068
P_1_T_2_	−10.61	0.000	0.90	0.407	1.40	0.220	−1.47	0.185	2.91	0.020	−0.56	0.589	1.57	0.168	−4.43	0.004
P_2_T_1_	−7.48	0.002	4.93	0.001	1.50	0.172	−1.77	0.121	2.17	0.062	0.56	0.592	1.86	0.112	−1.78	0.125
P_2_T_2_	−6.76	0.007	8.01	0.000	0.87	0.411	−1.12	0.305	2.06	0.092	0.19	0.857	0.72	0.499	−5.49	0.002
P_3_T_1_	−6.11	0.004	15.38	0.000	−0.48	0.644	−0.73	0.487	2.12	0.067	2.03	0.077	4.49	0.004	8.46	0.003
P_3_T_2_	−6.35	0.003	18.04	0.000	−1.92	0.092	−0.24	0.817	−0.45	0.665	−0.86	0.413	12.28	0.000	4.67	0.003

^1^ *T*-tests were performed to compare each treatment to the control (CK). t: t-statistic; p: significance level.

## Data Availability

The original contributions presented in this study are included in the article. Further inquiries can be directed to the corresponding author.
